# A highly pathogenic avian influenza virus H5N1 clade 2.3.4.4 detected in Samara Oblast, Russian Federation

**DOI:** 10.3389/fvets.2024.1244430

**Published:** 2024-02-08

**Authors:** Anastasia Glazunova, Elena Krasnova, Tatiana Bespalova, Timofey Sevskikh, Daria Lunina, Ilya Titov, Irina Sindryakova, Andrey Blokhin

**Affiliations:** ^1^Federal Research Center for Virology and Microbiology, Branch in Samara, Samara, Russia; ^2^Federal Research Center for Virology and Microbiology, Samara, Russia; ^3^Federal Research Center for Virology and Microbiology, Branch in Nizhny Novgorod, Nizhny Novgorod, Russia

**Keywords:** influenza A viruses, HPAIV, subtype virus, active monitoring, wild birds, Eurasian Teal, Russia

## Abstract

Avian influenza (AI) is a global problem impacting birds and mammals, causing economic losses in commercial poultry farms and backyard settings. In 2022, over 8,500 AI cases were reported worldwide, with the H5 subtype being responsible for many outbreaks in wild and domestic birds. In the territory of the Russian Federation, outbreaks of AI have been massively reported since 2020, both among domestic bird species and wild bird species. Wild migratory birds often serve as natural reservoirs for AI viruses, and interactions between bird species can lead to the emergence of new, highly pathogenic variants through genetic recombination between strains. In order to combat the widespread outbreaks of the disease and potential risks of further spread in 2021, monitoring studies were conducted in the Samara Oblast, the southeastern region of European Russian Federation. These studies aimed to diagnose and characterize circulating AI virus variants among wild migratory birds during waterfowl hunting in areas of mass nesting. Among the 98 shot birds, a highly pathogenic A/H5N1 AI virus was detected in a Eurasian Teal from the Bolshechernigovsky district. It was classified into clade 2.3.4.4 based on the cleavage site structure of HA. Phylogenetic analysis showed a high relatedness of the identified strain in the Samara Oblast with field isolates from Russia, Nigeria, Bangladesh, and Benin. The article emphasizes the importance of monitoring AI virus spread in both wild and poultry, highlighting the need for timely information exchange to assess risks. Further comprehensive studies are necessary to understand virus dissemination pathways.

## 1 Introduction

The global spread of avian influenza (AI) in the world (1, 2) poses a serious threat to animal and human health (3–5). Mass outbreaks of the disease result in the culling of millions of birds and significant economic losses, both on large farms and small holdings (6–9). The AI virus has been detected in a wide range of free-living birds (more than 75 species belonging to various orders). The virus has been found in representatives of the orders *Ciconiiformes, Anseriformes, Charadriiformes, Pelecaniformes, Podicipedidae, Gruiformes, Falconiformes*, and *Passeriformes* (10). Among these orders, wild waterfowl serve as the main reservoir of AI virus (11–13), and their role in virus transmission and maintenance of its epidemic cycle has been confirmed by numerous studies worldwide (14–16). Among all bird species, representatives of the orders *Anseriformes* and *Charadriiformes* play a particular role in maintaining the epidemiological cycle, carrying most subtypes of influenza A virus asymptomatically (17–19). Due to the segmented genome, influenza viruses are prone to reassortment and antigenic drift (20–22), leading to the emergence of new subtypes and strains capable of evading the host’s immunity and causing infection not only in birds, but also in many mammalian species (including humans) (23, 24). The detection of highly pathogenic avian influenza (HPAI) H5 viruses in wild and domestic birds in Europe often coincides with the autumn migration in the southwest/west and with large local aggregations of waterfowl during the wintering period (25). In recent years, clade 2.3.4.4 has become the dominant clade among all HPAI H5 viruses, further subdivided into subclades a–h (24).

Among all subtypes of influenza A virus, H5N1 stands out, as it has spread widely throughout Asia, Europe and Africa, a range of both domestic and wild bird species, and caused sporadic cases among humans and other mammals. This subtype is lethal to domestic poultry in 100% of cases (24). The largest number of HPAI H5N1 outbreaks among domestic and wild birds in the world was reported in 2004–2006 (1), and since 2021 to the present day (1, 6).

**Figure 1:**
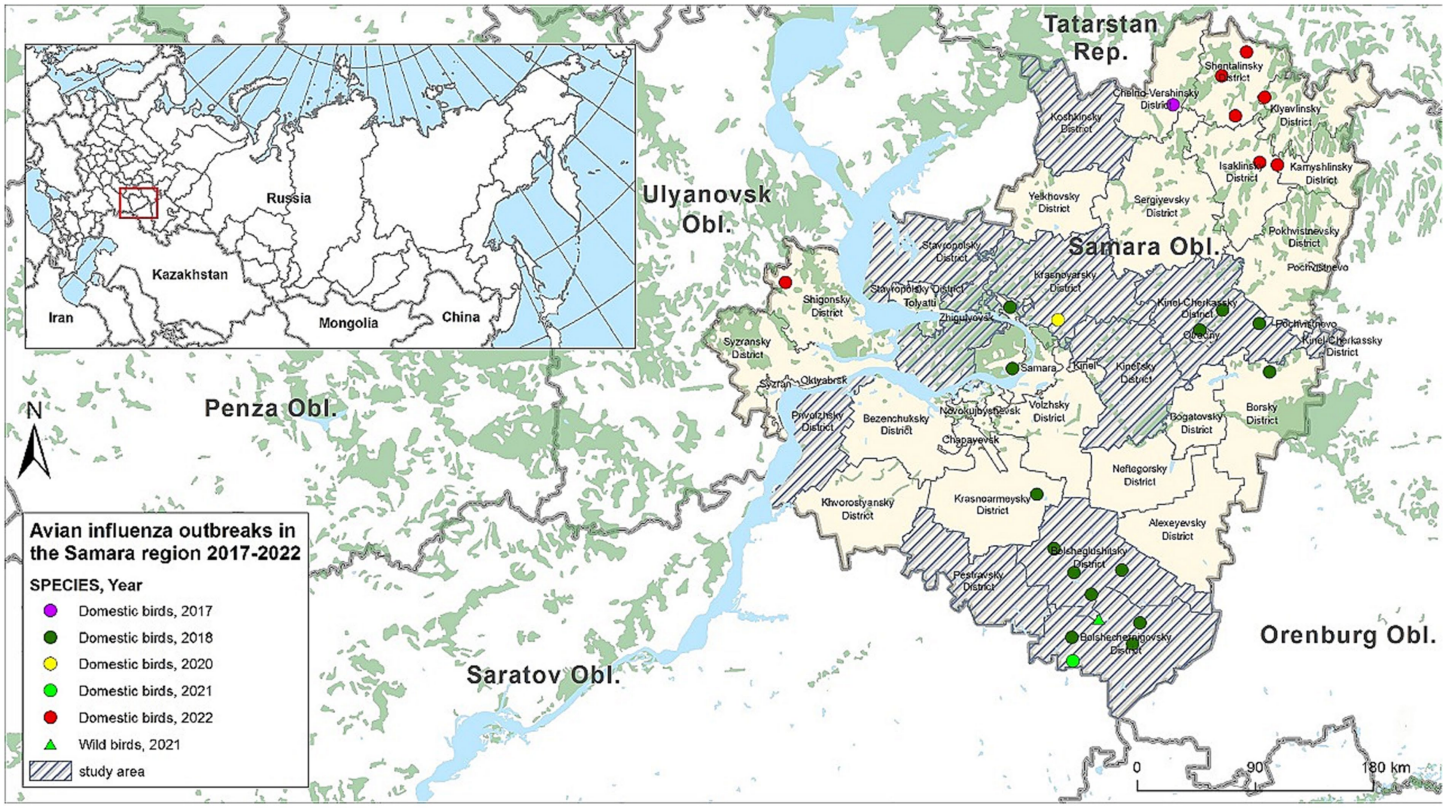
Outbreaks of AI in the Samara Oblast in 2017–2022 and areas where studies were conducted (ArcGIS Desktop 10.6.1).

In the territory of the Russian Federation (RF), HPAI was first identified on 27.07.2005, and until 2010, the H5N1 subtype was registered in the affected areas (26). After a lull and stabilization of the situation, the re-registration of outbreaks in the country occurred in 2014, and in addition to the previously circulating H5N1 subtype, the H5N8 subtype was detected in wild migratory birds, and in 2016, among domestic poultry. The widespread dissemination of the disease among both domestic poultry and in the wild occurred in Russia from 2020, with outbreaks associated with the H5N8 subtype, and in the second half of 2021, with the H5N1 subtype (27, 28). In the Samara Oblast—a region bordering Kazakhstan and part of the Central Asian flyway for birds—outbreaks of avian influenza have been registered annually since 2017 (Figure 1). Cases of influenza infection were associated both with the introduction of the virus with migratory birds and with illegal trade of young birds at markets and fairs in the region (29), as well as the export of infected poultry products to neighboring regions (30). The combination of average swampiness, the presence of lakes with a moderately continental climate (31) provides comfortable conditions for a variety of wild migratory bird species that serve as reservoirs and spreaders of the influenza virus. Considering these factors, Samara Oblast was selected for active avian influenza monitoring among wild birds during the hunting season.

The main goal of our study was to detect avian influenza virus in wild migratory birds. Samara Oblast, located in the southeast of the European part of the Russian Federation and intersected by the Central Asian flyway, was selected for monitoring studies. Overall, the aim of our work was to conduct monitoring studies to detect AI virus in wild bird populations, followed by determining the potential source of the identified virus through phylogenetic analysis based on data about HPAI strains isolated from wild birds in 2021.

## 2 Materials and methods

### 2.1 Research area and objects

Expeditionary field trips were conducted in collaboration with hunters from the Department of Hunting and Fishing of the Samara Oblast at locations of mass gathering of wild waterfowl. Monitoring studies were conducted during the 2021 hunting seasons for wild migratory and synanthropic birds. Bird hunting was carried out by hunters from the Department of Hunting and Fishing of the Samara Oblast in 9 administrative districts: Bolsheglushitsky, Bolshechernigovsky, Kinelsky, Kinel-Cherkassky, Koshkinsky, Krasnoyarsky, Pestrovsky, Privolzhsky, and Stavropolsky districts (Figure 1).

Within the framework of the study, randomly selected bird species were considered, with particular attention given to orders of birds in which the AI virus had been detected according to published data. Initially, the number of shot individuals was not established due to the inability to accurately determine their quantity, caused by the uncertainty of hunting outcomes.

### 2.2 Sample collection

Immediately after hunting, samples were taken from the birds using sterile surgical instruments and sterile containers. All samples were placed in sterile disposable plastic tubes of the “Eppendorf” type with transport medium, which were hermetically sealed and placed in safe bags. One safe bag contained tubes with biological material obtained from one individual.

The following pathological-anatomical material was taken from the hunted wild birds: lungs, trachea, intestinal tract, liver, spleen, kidneys, heart. In total, during the monitoring studies, pathoanatomical material was collected from 98 wild migratory birds.

Pathoanatomical material for research on AI was packed and transported in accordance with sanitary rules SanPiN 3.3686–21 (32). Sealed samples in a container with reusable cold elements were delivered to the laboratory with accompanying documents. All screening studies were conducted at the Federal Research Center of Virology and Microbiology (FRCVM).

To store and process data obtained during monitoring studies, a free mobile application for data collection, Epicollect5,^1^ was used. A project called “Avian influenza monitoring in Russia” was created on this platform. Using the available constructor in the program, a database containing the following information was created: the date of sample collection, the subject of the Russian Federation (region, republic), the administrative district, the date of the last HPAI outbreak in the administrative district, the name of the hunting ground where monitoring was carried out, the area of the hunting ground in hectares, geocoordinates, the type (active or passive) and period of monitoring, the species and sex of the hunted bird, the type of obtained samples, the barcode for each sample collected, the method of research, and the results obtained. For ease of use, the database was supplemented with a “Note” column, and the ability to produce and save photo materials was provided. All data could be easily exported in xlsx format. The application automatically displayed dynamic maps based on the coordinates of the research locations.

### 2.3 Screening studies and nucleotide sequencing

For study, samples of different organs from each examined bird were pooled into one sample. RNA pool isolation from pathological material was performed in accordance with “Instruction No. I-0158-01 for the detection of the genome of highly pathogenic avian influenza virus type A by real-time polymerase chain reaction” (FRCVM, 2021).

PCR was performed on the obtained RNA using the Superscript III One-Step RT-PCR System with Platinum Taq DNA Polymerase commercial kit (Thermo Fisher Scientific, Waltham, Massachusetts, United States): Real-time RT-PCR—detection of Influenza A Virus genome (M1.2) (33).

Primers developed at the Friedrich Loeffler Institut (Germany) were used for amplification of the HA (700 base pairs) and NA (650 base pairs) gene regions (34). The reaction mixture for HA and NA amplification consists of: RNase-freewater −4.5 μL; 2x reaction mix −12.5 μL; forward and reverse primers (10 μM) 1:1 μL; SSIII RT-/Platinum Taq Mix −1.0 μL; RNA sample 5 μL. Primer sequences, which is used for amplification of HA and NA fragments are presented (Supplementary Table S1). The temperature–time profile for RT-PCR amplification used was as follows: 10 min at 45°C, Taq activation for 10 min at 95°C, denaturation for 10 s at 95°C, annealing for 20 s at 56°C, extension for 30 s at 72°C, 45 cycles total.

Detection and confirmation of the molecular weight of PCR products were carried out on a 1.5% TAE agarose gel with 0.01% ethidium bromide as an intercalating dye.

Nucleotide sequencing was performed using the ABI 3130 Genetic Analyzer (Applied Biosystems, Foster City, CA, United States) and the Big Dye Terminator v3.1 Cycle Sequencing Kit (Applied Biosystems, Foster City, CA, United States) according to the manufacturer’s instructions. The same specific oligonucleotides used for PCR amplification were used for determining the nucleotide sequence of the target genes.

Parallel reactions with defective dideoxynucleotides were carried out with forward and reverse primers using the following thermal conditions: 95°C for 10 s, 50°C for 20 s, 60°C for 4 min, for a total of 30 cycles. The sequencing reaction mixture was purified using the Big Dye XTerminator Purification Kit (Applied Biosystems, Foster City, CA, United States) according to the manufacturer’s instructions.

### 2.4 Phylogenetic analysis

Nucleotide sequences obtained during the study were compared with known gene sequences of highly pathogenic avian influenza virus identified in the same year (2021) to assess potential viral spread during bird migration and reproduction. Comparison was conducted using the BLASTN^2^ (35) and MEGA11 (Molecular Evolutionary Genetics Analysis, version 11.0.13) (36) programs.

The clade was determined based on the cleavage site structure of the amino acid sequence HA (PLREKRRKR/GLF) in accordance with the guiding principles of the OIE (37).

All available known gene sequences of highly pathogenic avian influenza virus collected in 2021 and accessible in GenBank were considered. Sequences with a high percentage of homology were selected from all uploaded sequences. For sequences of NA segments, the maximum values were chosen, which were at least 99.4%, and for sequences of HA segments—at least 98.5%. Sequence identity was determined based on the statistical significance of matches in the BLASTN program (38).

**Figure 2:**
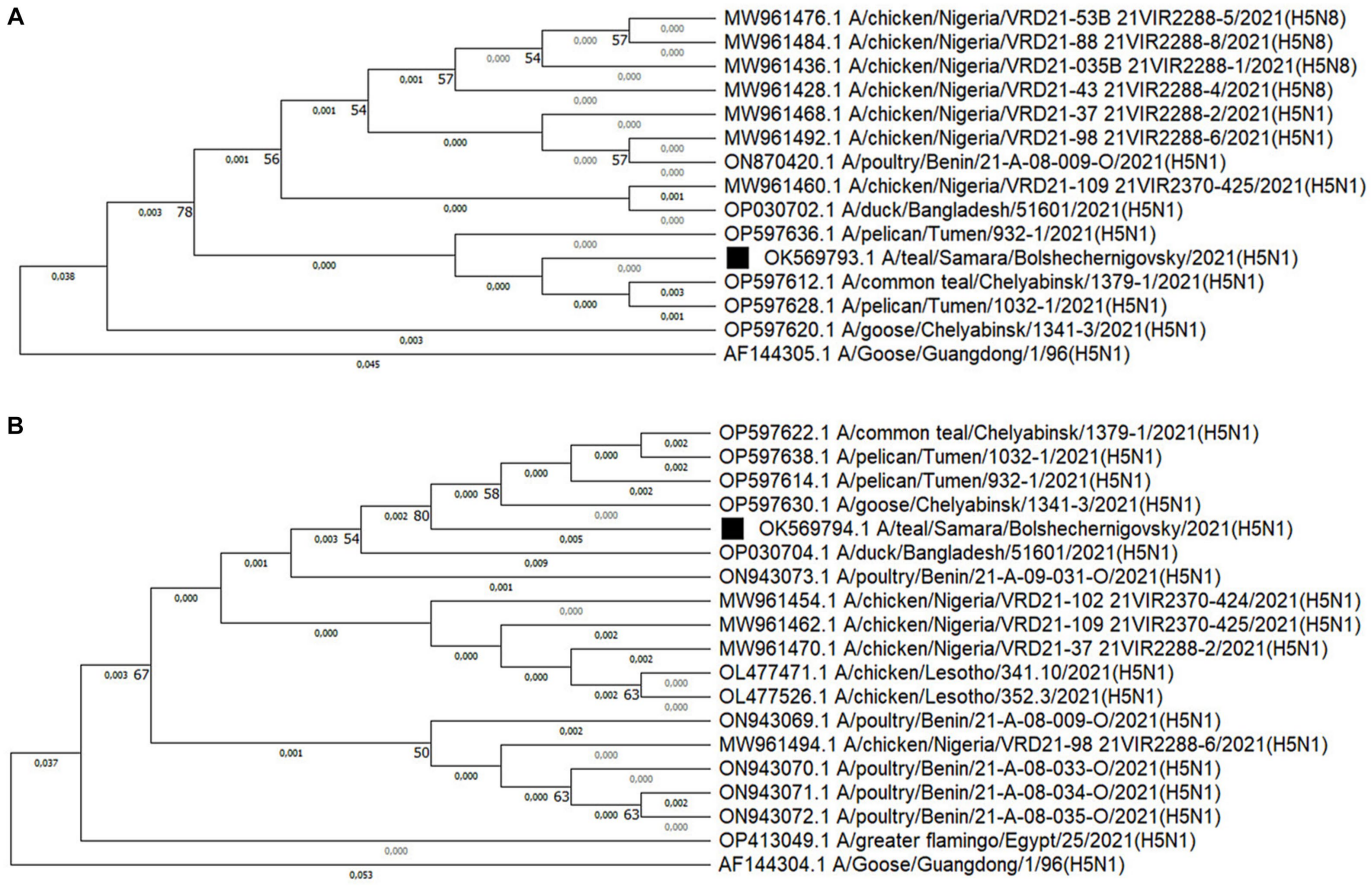
Phylogenetic dendrograms constructed from fragments of the HA gene **(A)** and the NA gene **(B)** using the neighbor-joining method with a bootstrap value of 1,000. The isolate obtained during active monitoring in the Samara Oblast is indicated by a black square and was assigned to clade 2.3.4.4 based on the amino acid sequence of HA. Additionally, the ancestor organism isolate was added to the phylogenetic dendrograms.

The alignment of nucleotide sequences was performed in the MEGA11 program with the Align by MUSCLE function. Evolutionary history was reconstructed using the neighbor-joining method (39) with bootstrap value of 1,000. The optimal tree was shown. The percentage of replicate trees in which the associated taxa clustered together in the bootstrap test (1,000 replicates) is shown next to the branches (40) (Figure 2). Evolutionary distances were calculated using the maximum composite likelihood method (41) and expressed in units of base substitutions per site. All ambiguous positions were removed for each sequence pair.

## 3 Results

### 3.1 Sample collection

As а part of avian influenza monitoring in the wild, bird shooting was conducted from April 15th to May 15th, 2021, in 9 municipal districts, and from mid-September to mid-October 2021 in 6 municipal districts of the Samara Oblast. Throughout the active AI monitoring period in the Samara Oblast, 41.8% (41 individuals) of the shot birds belonged to the order *Passeriformes*, including Crow (*Corvus corax*) at 14.6% (6 individuals), and Common magpie (*Pica pica*) and Starling (*Sturnus vulgaris*) at 7.3% each (3 individuals), Jackdaw (*Corvus monedula*) at 19.5% (8), Rook (*Corvus frugilegus*) at 51.2% (21), 22.5% (22) individuals from the order *Anseriformes* (Duck (*Anas platyrhynchos*) at 68.2% (15), Goose (*Anser anser*) and Teal (*Anas crecca*) at 4.55% each (1), Teal (*Spatulaquer quedula*) at 22.7% (5)), 15.3% (15) individuals from the order *Charadriiformes* [Lapwing (*Vanellus vanellus*)—13.3% (2) and Seagull (*Chroicocephalus ridibundus*) at 86.7% (13)], 8.2% (8) individuals from the order *Columbiformes* (Pigeons (*Columba livia*)), 7.1% (7) individuals from the order *Pelecaniformes* (Heron (*Ardea cinerea*)), 4.1% (4) individuals from the order *Suliformes* (Cormorant (*Phalacrocorax carbo*)), and 1% (1) individual from the order *Podicipediformes* (Chomga (*Podiceps cristatus*)). During the hunting period from September to October, 60.2% (59) birds were hunted, with the highest percentage being Rook (*Corvus frugilegus*) (16.9%), as in the spring period, as well as Seagull (*Chroicocephalus ridibundus*) (15.3%) and Pigeons (*Columba livia*) (13.6%) (Supplementary Table S2). The collection, storage, transmission, and processing of epidemiological data were carried out using the Epicollect5 program. The software was tested by both the authors and hunters on smartphones. It proved to be easy to learn and significantly simplified and accelerated the process of compiling and mapping reports on sample collection in the wild.

### 3.2 Screening studies and nucleotide sequencing

In specimens from 98 birds, AI virus subtype A (Avian Influenza A Virus) was detected in samples from one Eurasian teal (*Anas crecca*). No visual pathological anatomical changes were detected during the autopsy of this specimen. Nucleotide sequence of the HA and NA genes fragment were obtained: 701 bp for HA and 577 bp for NA. Based on the analysis of the NA and HA sequences, the detected HPAI virus was identified as H5N1. This isolate was named A/teal/Samara/Bolshechernigovsky/2021(H5N1). The HA and NA sequences were deposited in GenBank under accession numbers OK569793.1 and OK569794.1, respectively (42).

Molecular genetic methods revealed that the HA protein of the studied isolate has a multi-basic cleavage site sequence PLREKRRKR/GLF, which is typical for H5 HPAI viruses of the Goose/Guangdong clade 2.3.4.4 lineage (Asia, Africa, Europe, and North America) (43), which corresponds to Clade 2.3.4.4 in modern nomenclature (44).

### 3.3 Phylogenetic analysis

Thirteen homologous isolates were found in the BLASTN database (38) for the HA gene, and 17 for the NA gene. The degree of homology ranged from 99.43 to 100.00% for HA (Table 1) and from 98.61 to 99.65% for NA (Table 2).

**Table 1:** Isolates with a high degree of homology by HA A/teal/Samara/Bolshechernigovsky/2021(H5N1) (GenBank: OK569793.1).

Number in GenBank	Sampling year	Country	Name of the strain	% match
OP597636.1	2021	Russian Federation	Influenza A virus [A/pelican/Tumen/932–1/2021(H5N1)] segment 4 hemagglutinin (HA) gene, complete cds	100.00%
OP597628.1	2021	Russian Federation	Influenza A virus [A/pelican/Tumen/1032–1/2021(H5N1)] segment 4 hemagglutinin (HA) gene, complete cds	99.86%
OP030702.1	2021	People’s Republic of Bangladesh	Influenza A virus [A/duck/Bangladesh/51601/2021(H5N1)] segment 4 hemagglutinin (HA) gene, complete cds	99.86%
OP597612.1	2021	Russian Federation	Influenza A virus [A/common teal/Chelyabinsk/1379–1/2021(H5N1)] segment 4 hemagglutinin (HA) gene, complete cds	99.71%
ON870420.1	2021	Republic of Benin	Influenza A virus [A/poultry/Benin/21-A-08-009-O/2021(H5N1)] segment 4 hemagglutinin (HA) gene, complete cds	99.71%
MW961492.1	2021	Federal Republic of Nigeria	Influenza A virus [A/chicken/Nigeria/VRD21-98_21VIR2288-6/2021(H5N1)] segment 4 hemagglutinin (HA) gene, complete cds	99.71%
MW961468.1	2021	Federal Republic of Nigeria	Influenza A virus [A/chicken/Nigeria/VRD21-37_21VIR2288-2/2021(H5N1)] segment 4 hemagglutinin (HA) gene, complete cds	99.71%
MW961460.1	2021	Federal Republic of Nigeria	Influenza A virus [A/chicken/Nigeria/VRD21-109_21VIR2370-425/2021(H5N1)] segment 4 hemagglutinin (HA) gene, complete cds	99.71%
MW961484.1	2021	Federal Republic of Nigeria	Influenza A virus [A/chicken/Nigeria/VRD21-88_21VIR2288-8/2021(H5N8)] segment 4 hemagglutinin (HA) gene, complete cds	99.71%
MW961476.1	2021	Federal Republic of Nigeria	Influenza A virus [A/chicken/Nigeria/VRD21-53B_21VIR2288-5/2021(H5N8)] segment 4 hemagglutinin (HA) gene, complete cds	99.71%
MW961436.1	2021	Federal Republic of Nigeria	Influenza A virus [A/chicken/Nigeria/VRD21-035B_21VIR2288-1/2021(H5N8)] segment 4 hemagglutinin (HA) gene, complete cds	99.71%
MW961428.1	2021	Federal Republic of Nigeria	Influenza A virus [A/chicken/Nigeria/VRD21-43_21VIR2288-4/2021(H5N8)] segment 4 hemagglutinin (HA) gene, complete cds	99.71%
OP597620.1	2021	Russian Federation	Influenza A virus [A/goose/Chelyabinsk/1341–3/2021(H5N1)] segment 4 hemagglutinin (HA) gene, complete cds	99.43%

**Table 2:** Isolates with a high degree of homology by NAA/teal/Samara/Bolshechernigovsky/2021(H5N1) (GenBank: OK569794.1).

Number in GenBank	Sampling year	Country	Name of the strain	% match
OP597622.1	2021	Russian Federation	Influenza A virus [A/goose/Chelyabinsk/1341–3/2021(H5N1)] segment 6 neuraminidase (NA) gene, complete cds	99.65%
OP597638.1	2021	Russian Federation	Influenza A virus [A/pelican/Tumen/932–1/2021(H5N1)] segment 6 neuraminidase (NA) gene, complete cds	99.48%
OP597630.1	2021	Russian Federation	Influenza A virus [A/pelican/Tumen/1032–1/2021(H5N1)] segment 6 neuraminidase (NA) gene, complete cds	99.48%
OP597614.1	2021	Russian Federation	Influenza A virus [A/common teal/Chelyabinsk/1379–1/2021(H5N1)] segment 6 neuraminidase (NA) gene, complete cds	99.48%
MW961454.1	2021	Federal Republic of Nigeria	Influenza A virus [A/chicken/Nigeria/VRD21-102_21VIR2370-424/2021(H5N1)] segment 6 neuraminidase (NA) gene, complete cds	99.13%
ON943073.1	2021	Republic of Benin	Influenza A virus [A/poultry/Benin/21-A-09-031-O/2021(H5N1)] segment 6 neuraminidase (NA) gene, complete cds	98.96%
ON943072.1	2021	Republic of Benin	Influenza A virus [A/poultry/Benin/21-A-08-035-O/2021(H5N1)] segment 6 neuraminidase (NA) gene, complete cds	98.96%
ON943070.1	2021	Republic of Benin	Influenza A virus [A/poultry/Benin/21-A-08-033-O/2021(H5N1)] segment 6 neuraminidase (NA) gene, complete cds	98.96%
OL477526.1	2021	Kingdom of Lesotho	Influenza A virus [A/chicken/Lesotho/352.3/2021(H5N1)] segment 6 neuraminidase (NA) gene, complete cds	98.96%
OL477471.1	2021	Kingdom of Lesotho	Influenza A virus [A/chicken/Lesotho/341.10/2021(H5N1)] segment 6 neuraminidase (NA) gene, complete cds	98.96%
MW961494.1	2021	Federal Republic of Nigeria	Influenza A virus [A/chicken/Nigeria/VRD21-98_21VIR2288-6/2021(H5N1)] segment 6 neuraminidase (NA) gene, complete cds	98.96%
MW961470.1	2021	Federal Republic of Nigeria	Influenza A virus [A/chicken/Nigeria/VRD21-37_21VIR2288-2/2021(H5N1)] segment 6 neuraminidase (NA) gene, complete cds	98.96%
MW961462.1	2021	Federal Republic of Nigeria	Influenza A virus [A/chicken/Nigeria/VRD21-109_21VIR2370-425/2021(H5N1)] segment 6 neuraminidase (NA) gene, complete cds	98.96%
ON943071.1	2021	Republic of Benin	Influenza A virus [A/poultry/Benin/21-A-08-034-O/2021(H5N1)] segment 6 neuraminidase (NA) gene, complete cds	98.78%
ON943069.1	2021	Republic of Benin	Influenza A virus [A/poultry/Benin/21-A-08-009-O/2021(H5N1)] segment 6 neuraminidase (NA) gene, complete cds	98.78%
OP413049.1	2021	The Arab Republic of Egypt	Influenza A virus [A/greater flamingo/Egypt/25/2021(H5N1)] segment 6 neuraminidase (NA) gene, partial cds	98.74%
OP030704.1	2021	People’s Republic of Bangladesh	Influenza A virus [A/duck/Bangladesh/51601/2021(H5N1)] segment 6 neuraminidase (NA) gene, complete cds	98.61%

Sequence alignment, neighbor-joining method phylogenetic analysis, and tree construction were performed using MEGA11 software (36). For homology analysis, 15 nucleotide sequences were used, out of which 13 were selected based on a high degree of homology. Additionally, the ancestral organism (GenBank under accession numbers AF144305.1) and the isolated strain were included in the analysis (Figure 2A). All ambiguous positions were removed for each sequence pair (42).

The HA sequence of the A/teal/Samara/Bolshechernigovsky/2021(H5N1) isolate was homologous to the HA sequences of isolates from the Urals Federal District of Russia [Tyumen (99.87 and 100%) and Chelyabinsk Oblasts (99.43 and 99.71%)], West Africa [Nigeria (99.71%), Benin (99.71%)], and South Asia (Bangladesh by 99.87%) deposited in Genbank.

Nineteen nucleotide sequences were selected for NA analysis, out of which 17 were selected based on a high degree of homology. Additionally, the ancestral organism (GenBank under accession numbers AF144304.1) and the isolated strain were included in the analysis (Figure 2B). The tree was drawn to scale, with branch lengths measured in the number of substitutions per site. The NA sequence of the A/teal/Samara/Bolshechernigovsky/2021 (H5N1) isolate was homologous to the NA sequences of isolates from the Urals Federal District of Russia (Tyumen (99.48%) and Chelyabinsk (99.48 and 99.65%) Oblasts), West [Nigeria (98.96 and 99.13%), Benin (98.78 and 98.96%)], South (Lesotho by 98.96%), and North Africa (Egypt by 98.74%), and South Asia (Bangladesh by 98.61%) deposited in Genbank.

## 4 Discussion

The global spread of HPAI outbreaks was reported in 2021–2022. During this period, 3,573 cases of HPAI in wild birds were detected in European countries (45). Studies conducted in Sweden (46) and in Norway (47) confirmed that the introduction of the virus into the country most likely occurred through migratory waterfowl, which is a confirmed risk factor for the cross-border spread of HPAI. Within countries and on their bordering territories, the expansion of the virus is often associated with violations of biosecurity measures in breeding domestic poultry (48). Similar factor was precisely the reason for the spread of AI in the Samara Oblast from 2017 to 2021 (29, 30). At the same time, the avifauna of the Samara Oblast is characterized by species diversity and is represented by 293 bird species (31). The region is entering а zone of the Central Asian flyway, which creates conditions for the introduction of infections with migratory birds (31).

As a result of monitoring in 2021 in the Samara Oblast the genome of AI virus was detected in a Eurasian teal, which was collected near a settlement. The regional veterinary service imposed a quarantine in the hunting ground (where monitoring studies were conducted) and establish the boundaries of the outbreak. The neighboring settlement (Pekilyanka village) entered the threatened zone, where containment and quarantine measures were also carried out. Additionally, a prohibition was enforced on the treatment of sick domestic birds, outdoor poultry farming, transportation of domestic birds, as well as the export and sale of poultry products and feed. All containment and quarantine measures were conducted in accordance with the order of the Ministry of Agriculture of the Russian Federation dated March 24, 2021, No. 158 “On approval of the Veterinary Rules for the implementation of preventive, diagnostic, restrictive, and other measures, establishment and cancelation of quarantine and other restrictions aimed at preventing the spread and elimination of outbreaks of highly pathogenic avian influenza” (49). Considering the absence of large poultry farms in the settlement, all personal subsidiary farms engaged in poultry farming for personal needs were subject to containment measures.

It is worth noting that the Eurasian teal is one of the most common species of the duck family in Europe and Russia (50, 51). After breeding in Siberia and Northern Europe, this species migrates in August–September to spend winter in Western Europe. In February, it begins spring migration from southern wintering grounds (52). The Eurasian teal can cover long distances (an average of 160 km over 30 days) and is one of the main natural reservoirs of AI virus (53), and therefore may have a high potential for spreading this virus (54). The following subtypes of AI have been isolated from Eurasian teals in different countries: H12N3, H8N4, H15N4, N5N1 (52, 55–60).

It has been identified that the AI virus found in the Eurasian teal belongs to clade 2.3.4.4 according to the characteristics of the HA cleavage site region and is classified as A/H5N1. It is known that global spread of clade 2.3.4.4 AI has been registered since 2016 in 48 countries in Asia, Africa, North America and Europe (61). According to research by the FGBI “ARRIAH” and supplemented GISAID databases, it was established that AI strains isolated in Russia in 2021, based on the nucleotide sequence of the NA gene, belonged to genetic clade 2.3.4.4 (62). Subtypes H5N8, H5N6, and H5N5 were more commonly notified from clade 2.3.4.4, but they have gradually been displaced by the H5N1 virus subtype, which has been circulating in Europe since 2020 (63).

To study the origin of the HPAI virus isolate detected during monitoring studies in wild bird populations in 2021, a phylogenetic analysis of two genome segments (NA and HA) was performed, which allowed for the identification of a possible source of introduction. The analysis revealed that the HPAI virus isolated in the Samara Oblast was closely related (by HA segment) to H5N1 viruses isolated in Russia (Tyumen Oblast by 99.86–100%) and in South Asia (Bangladesh by 99.86%) in 2021. Additionally, the identified isolate showed similarities to viruses found in Russia (Chelyabinsk Oblast by 99.71 and 99.43%) and African countries (Nigeria, Benin, Lesotho, Egypt by 99.71%) during the same period, albeit to a lesser extent. High homology was observed with isolates from Russia, particularly with influenza A/pelican virus/Tyumen/2021(H5N1) (GenBank: OP597636.1, OP597638.1) and Influenza A virus [A/duck/Bangladesh/51601/2021(H5N1)] (GenBank: OP030702.1) (Table 1). The HPAI virus isolated in the Samara Oblast was closely related (by NA segment) to H5N1 viruses isolated in Russia (Chelyabinsk and Tyumen Oblasts by 99.48 and 99.65% respectively) in 2021. Additionally, the identified isolate showed similarities to viruses found in South Asia (Bangladesh by 98.61%) and African countries (Nigeria, Benin, Lesotho, Egypt by 99.13, 98.96, 98.78, and 98.74% respectively) during the same period, albeit to a lesser extent. High homology was observed with isolates from Russia, particularly with Influenza A virus isolates from Chelyabinsk [A/goose/Chelyabinsk/2021(H5N1)] (GenBank: OP597622.1, OP597614.1) and Tyumen regions [A/pelican/Tyumen/2021(H5N1)] (GenBank: OP597638.1, OP597630.1) (Table 2). It is worth noting that for both segments, the Russian isolates exhibited a high degree of identity in nucleotide sequence in the HA segment (99.43–100.00%) and the NA segment (99.48–99.65%).

Based on the obtained data and the high similarity with isolates from the Tyumen Oblast, it should be noted that there was a report in the mass media about the mass mortality of Dalmatian pelicans (*Pelecanus crispus*) at their breeding site on Lake Cherno. More than 100 adult birds were found dead, but no deaths of pelican chicks or other waterfowl species that cohabit with them were recorded. Real-time PCR analysis of samples from five of the dead birds revealed the presence of subtype A (Avian Influenza A Virus)—H5 (64).

The high homology of the identified isolate with strains from West Africa (Nigeria, Benin) may indicate the circulation of the virus in the populations of waterfowl of West Africa south of the Sahara, where wetland birds’ winter from October to April from Eurasia. Thus, wild birds in West Africa may serve as reservoirs for maintaining AI during the winter when the disease is rarely encountered in wintering waterfowl in Europe. These territories are also ecosystems with favorable conditions for the emergence of new virus subtypes as a result of genetic mutations and reassortment AI viruses of different geographic origins (65).

Since January 2021, outbreaks caused by HPAI H5N1 clade 2.3.4.4b have been reported in many West African countries (66). There is evidence of virus spread among neighboring countries in West Africa due to anthropogenic factors. For example, the Influenza A virus [A/poultry/Benin/21-A-08-009-O/2021(H5N1)] isolate was obtained from dead chicks at poultry farms in the Sem-Podji and Wida provinces in southern Benin in August–September 2021. This isolate was closely related to H5N1 viruses isolated in Nigeria in 2021, which likely indicates a common ancestor and suggests a possible transmission route from Nigeria to Benin. It is quite possible that the introduction of the H5N1 virus into Benin was due to the movement of infected poultry and by-products from neighboring Nigeria. In fact, the detection of closely related isolates in Nigeria in 2021 coincided with the period (January–March) when Eurasian migratory birds are present in West Africa, which supports the hypothesis of a possible initial introduction of H5N1 viruses into Africa due to bird migration (67).

Regarding the isolate from Bangladesh, it is known that (HPAI) H5N1 and low pathogenic avian influenza (LPAI) H9N2 viruses have caused significant damage to poultry farming in Bangladesh (68). Most H5N1 and H9N2 isolates were detected in live bird markets and in Tangail-Haor, in birds living in the wetlands area of Bangladesh where domestic ducks often come into contact with migratory birds. The study (69) mentions a high probability of interspecies transmission, genetic recombination of viruses, and the possibility of introduction of the virus by migratory birds from the Central Asian flyway. Further comprehensive research is required to understand the pathways of virus introduction, specifically the identity of the field isolate A/teal/Samara/Bolshechernigovsky/2021(H5N1) with the field isolate Influenza A virus A/duck/Bangladesh/51601/2021(H5N1).

As results on the obtained data and phylogenetic analysis, the infection of the Eurasian teal could have occurred both on the territory of the region and during migration through neighboring regions or other countries. Factors such as the duration of virus excretion and its inactivation rate in water, which were experimentally modeled in a study by Lebarbenchon et al. (52), may affect the spread of avian influenza virus with wild birds. Climatic and seasonal characteristics of the area, as well as the number of contacts with other bird species during migratory stops and the number of birds in flocks during migration, may significantly affect virus spread. Monitoring studies, both active and passive, are necessary for early detection of the virus in wild bird populations. Despite the advantages and disadvantages of each type of monitoring, it is worth noting the benefits of active monitoring studies. Active monitoring studies using hunted birds can provide a higher chance of virus detection and be more efficient tool in prediction of HPAI introduction into country than passive monitoring studies and can also be combined with routine hunter activity during seasonal bird hunting, when migration process is the most intense (70).

As shown in recent EFSA report (71), active monitoring of HPAI in wild birds during migration periods may help to predict virus spread 2–3 months prior to outbreaks. Also, active monitoring can be more effective tool for virus control than passive, as some may effectively survive infection and serve as reservoir for weeks (72). Studies on intra-and intercontinental spread of H5N1 avian influenza viruses among bird hosts are extremely important for reducing the global impact of viruses, controlling the risk of zoonotic diseases, and ensuring food security. Improving understanding of the pathways of circulation of avian influenza viruses in populations migratory birds will help provide early warning of the threat of introduction AI, which is crucial for the health of domestic birds and possibly humans. Although this brief overview is limited to a single case of AI virus detection and is not the result of long-term monitoring studies. Another limitation of the study was the impossibility of carrying out full genome sequencing of the obtained sample, which would have made it possible to more accurately analyze its origin and phylogenetic relationships with other strains. Despite this, the data obtained during the work are of great importance for exchanging information between countries and furthering global understanding of virus spread and evolution, which helps in early detection of viruses in the wild.

## Data availability statement

The datasets presented in this study can be found in online repositories. The names of the repository/repositories and accession number(s) can be found in the article/Supplementary material.

## Ethics statement

An ethical review was not required for this non-interventional study, according to local and national legislation. This article does not contain any studies involving animals performed by any of the authors.

## Author contributions

AG, EK, and AB: conceptualization. AG, EK, AB, IT, and TS: writing-original draft preparation. DL: visualization. AG and DL: conducting active monitoring in the wild and data collection. TB and IS: primary screening. IT: genotyping. All authors have read and agreed to the published version of the manuscript.

## Footnotes

^1^ https://five.epicollect.net/

^2^ http://blast.ncbi.nlm.nih.gov/Blast.cgi
