# Supplementary material for: A highly pathogenic avian influenza virus H5N1 clade 2.3.4.4 detected in Samara Oblast, Russian Federation
Source: Front Vet Sci. 2024 Feb 8;11:1244430. doi: 10.3389/fvets.2024.1244430 (PMC10881870; doi:10.3389/fvets.2024.1244430)
Supplement: Supplementary file 1 [file Data_Sheet_1.docx]

*Supplementary material*

A highly pathogenic avian influenza virus H5N1 clade 2.3.4.4 detected in Samara Oblast, Russian Federation.

Anastasia Glazunova^*^, Elena Krasnova, Tatiana Bespalova, Timofey Sevskikh, Daria Lunina, Ilya Titov, Irina Sindryakova and Andrey Blokhin

***Correspondence:**Anastasia Glazunova
GlazunovaAA@outlook.com

Table S1. Composition of the reaction mixture used for the amplification of NA and HA fragments

| Components of NA | 1 x | | |
| --- | --- | --- | --- |
| RNase free water | 4.5 µl | | |
| 2x reaction mix | 12.5 µl | | |
| AIV-886.1-F+AIV-886.2-F | 1.0 µl | | |
| AIVR_1458-R | 1.0 µl | | |
| SSIII RT-/Platinum Taq Mix | 1.0 µl | | |
| total: | 20.0 µl | | |
| Samples: 5µl RNA / reaction | | | |
| Components of HA | | 1 x |  |
| RNase free water | | 4.5 µl |  |
| 2x reaction mix | | 12.5 µl |  |
| HA-1057.1-F + HA-1057.2-F + HA-1057.3-F | | 1.0 µl |  |
| Bm-HA-R | | 1.0 µl |  |
| SSIII RT-/Platinum Taq Mix | | 1.0 µl |  |
| total: | | 20.0 µl |  |

Table S2. Summary data on shot game in the framework of active surveillance on avian influenza

| Species | Number of shot birds | | | | | | | | |
| --- | --- | --- | --- | --- | --- | --- | --- | --- | --- |
|  | Bolsheglushitsky district | Bolshechernigovsky district | Kinelsky district | Kinel-Cherkassky district | Koshkinsky district | Krasnoyarsky district | Pestrovsky district | Privolzhsky district | Stavropolsky district |
| *Chomga (Podiceps cristatus)* |  |  |  |  |  |  |  | 1 |  |
| *Cormorant (Phalacrocorax carbo)* | 1 | 1 | 2 |  |  |  |  |  |  |
| *Crow (Corvus corax)* | 2 |  | 1 | 1 | 1 |  |  |  | 1 |
| *Duck (Anas platyrhynchos)* | 2 | 3 |  | 4 |  | 4 | 1 | 1 |  |
| *Forty (Pica pica)* | 3 |  |  |  |  |  |  |  |  |
| *Goose (Anser anser)* | 1 |  |  |  |  |  |  |  |  |
| *Heron (Ardea cinerea)* |  | 5 |  |  |  |  | 1 | 1 |  |
| *Jackdaw (Corvus monedula)* | 3 |  |  |  | 3 | 1 |  |  | 1 |
| *Lapwing (Vanellus vanellus)* |  | 2 |  |  |  |  |  |  |  |
| *Pigeons (Columba livia)* | 3 |  |  | 5 |  |  |  |  |  |
| *Rook (Corvus frugilegus)* | 10 |  |  | 9 |  |  | 1 |  | 1 |
| *Seagull (Chroicocephalus ridibundus)* | 2 | 5 | 1 |  |  |  | 1 | 1 | 3 |
| *Starling (Sturnus vulgaris)* |  |  |  |  | 3 |  |  |  |  |
| *Teal (**Anas crecca)* |  | 1 |  |  |  |  |  |  |  |
| *Teal (Spatulaquer quedula)* |  | 1 |  | 1 |  | 2 |  | 1 |  |
| Total: | 27 | 18 | 4 | 20 | 7 | 7 | 4 | 5 | 6 |
